# Evaluation of the Safety and Efficacy of Hand Sanitizer Products Marketed to Children Available during the COVID-19 Pandemic

**DOI:** 10.3390/ijerph192114424

**Published:** 2022-11-03

**Authors:** Lauren E. Gloekler, Elise J. de Gandiaga, Natalie R. Binczewski, Katie G. Steimel, Andrey Massarsky, Jordan Kozal, Melissa Vincent, Rachel Zisook, Mark J. LaGuardia, Scott Dotson, Shannon Gaffney

**Affiliations:** 1 Stantec (ChemRisk), Aliso Viejo, CA 92656, USA; 2 Stantec (ChemRisk), San Francisco, CA 94104, USA; 3 Stantec (ChemRisk), Cincinnati, OH 45242, USA; 4 Virginia Institute of Marine Science, William & Mary, Gloucester Point, VA 23062, USA; 5 Insight Exposure and Risk Sciences Group, Cincinnati, OH 45249, USA

**Keywords:** hand sanitizer, consumer products, children, impurities, ethanol, COVID-19

## Abstract

Hand sanitizer use in the United States (U.S.) increased after the SARS-CoV-2 outbreak. The U.S. Food and Drug Administration (FDA) released temporary manufacturer guidance, changing impurity level limits for alcohol-based hand sanitizers (ABHSs). Since the guidance took effect, the FDA has recommended against using these hand sanitizers due to concerns over safety, efficacy, and/or risk of incidental ingestion. To address current gaps in exposure characterization, this study describes a survey of ABHSs marketed to children available in the U.S., as defined by several inclusion criteria. A subset of ABHSs (*n* = 31) were evaluated for ethanol and organic impurities using a modified FDA method. Products with detectable impurity levels were compared to the FDA’s established interim limits. Seven children’s products had impurity levels exceeding the FDA’s recommended interim limits, including benzene (up to 9.14 ppm), acetaldehyde (up to 134.12 ppm), and acetal (up to 75.60 ppm). The total measured alcohol content ranged from 52% to 98% in all hand sanitizers tested, ranging from 39% below, and up to 31% above, the labeled concentration. Future studies should confirm impurity contamination sources. A risk assessment could determine whether dermal application or incidental ingestion of impurity-containing hand sanitizers pose any consumer risk.

## 1. Introduction

**Figure 1:**
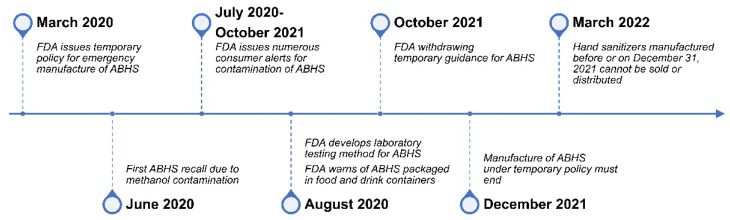
Timeline on Alcohol-Based Hand Sanitizer (ABHS)-Related U.S. Food and Drug Administration (FDA) Actions and Policies.

In 2019, the emergence of the new coronavirus SARS-CoV-2 resulted in increased sales and use of alcohol-based hand sanitizer (ABHS) because of concern of virus spread from contaminated hand surfaces. Increased demand for ABHSs from the general public and healthcare workers, however, led to difficulties accessing raw materials used in manufacturing, such as ethanol [1]. Hand sanitizers are classified as over-the-counter (OTC) drugs, and companies that manufacture these products are regulated by the U.S. Food and Drug Administration (FDA) [2]. To alleviate ABHS supply chain issues, the FDA allowed additional manufacturing entities that were not currently regulated as drug manufacturers to produce hand sanitizers, as long as the Secretary of Health and Human Services (HHS) declared a public health emergency [3]. To outline current manufacturing requirements for hand sanitizer production, the FDA promulgated several guidance documents [2,3,4] (Figure 1). These guidance documents provided temporary policies for preparing and manufacturing ABHS, as well as guidance for testing ethanol for contamination [5].

Per the FDA guidelines, hand sanitizers should be manufactured with no less than 94.9% by volume (*v*/*v*) ethanol or isopropyl alcohol, in addition to glycerin, hydrogen peroxide, and sterile water; the final formulation should have at least 80% ethanol (*v*/*v*) or 75% isopropyl alcohol (*v*/*v*) in an aqueous solution [3,6]. The FDA temporarily specified that fuel or technical grade ethanol were deemed acceptable for use in hand sanitizers. Regardless of the source, however, the ethanol should meet the following guidelines: (a) produced using fermentation and distillation processes “typically used for consumable goods” and contains no additives or additional chemicals; (b) meets United States Pharmacopoeia (USP) or Food Chemical Codex (FCC) grade requirements (including testing for impurities); and (c) screened for additional impurities not specified by USP and FCC requirements [3] (p. 7) [4,7]. Both the FDA temporary guidance and the World Health Organization (WHO) guidance state that hand sanitizers should not contain other active or inactive ingredients, such as additives, gelling agents, or fragrances to improve the smell or taste of the product, specifically “due to the risk of accidental ingestion in children” [4,8].

Impurities may potentially be introduced to hand sanitizer formulations through utilization of ethanol produced from fuel or technical grade ethanol via fermentation and distillation, or via the manufacturing environment (e.g., equipment and containers) [3]. Technical grade ethanol can be produced from a variety of feedstock, such as corn, sugar cane, sugar beet, wheat, and barley, and can contain byproducts and impurities that can potentially produce unpleasant odors or flavors and/or cause adverse human health effects [9]. Interim limits for specific impurities in ethanol have been provided for several compounds, and if the concentration of these in ethanol exceeds a sum of 300 ppm, then they must also meet interim limits for additional impurities [3]. In August 2020, FDA developed analytical procedures in support of its guidance, providing a methodology for manufacturers to help assure hand sanitizer products contain accurately labeled ingredients, and a screen for potentially harmful levels of impurities. The method provides guidance for detecting the impurities quantitatively or by using a “limit test approach”, in which an impurity is either greater or less than the corresponding peak [10]. Impurities in alcohol products, including methanol, acetates, aldehydes, butanols, amyl alcohols, propanols, and pentanols, are generated during grain fermentation. These compounds can form azeotropes that are co-distilled with the ethanol fraction, making purifying ethanol difficult [3,11].

Throughout the pandemic, the FDA tested and issued alerts for several hand sanitizer products from domestic and international manufacturers and identified products with unacceptable levels of methanol, 1-propanol, benzene, acetaldehyde, and acetal, and “concerningly low levels of ethyl alcohol or isopropyl alcohol” [12]. As of 10 August 2021, 255 hand sanitizer products were listed on the do-not-use list, the majority of which were manufactured in Mexico (*n* = 201) [12]. Other countries with products recalled or listed were China (*n* = 22), Guatemala (*n* = 6), Turkey (*n* = 3), Poland (*n* = 3), Korea (*n* = 1), and the U.S. (*n* = 13). Additionally, alerts were issued for hand sanitizers packaged in containers that resembled food pouches, water bottles, juice bottles, vodka bottles, or beer cans, as well as those containing food flavors, as these products may lead to incidental hand sanitizer ingestion, especially in children [12]. These containers were also labeled with children’s characters or movie references, including Barbie, Hot Wheels, Paw Patrol, Minions, JoJo Siwa, and Trolls World Tour [12].

Some hand sanitizers have particular characteristics (e.g., colorful or food-like packaging, cartoon characters, or candy and fruit scents) that may make them more attractive to children [13]. Children represent a susceptible group who may be at an increased risk of negative impacts from hand sanitizer impurities. Hand sanitizers are intended to be applied to hand surfaces; dermal contact with potential impurities is therefore likely the most common consumer exposure route. Intentional (as a result of substance abuse or mental health issues) and unintentional (accidental) ingestion of alcohol-based hand sanitizer in adults and children, however, does occur [14,15,16]. Increased exposure to hand sanitizers, including ingestion, was reported in children 12 years old and younger during the COVID-19 pandemic [2,17].

While inhalation of compounds present in hand sanitizers is possible, inhalation would not be expected to represent a primary exposure route relative to dermal and ingestion exposures, based on the National Poison Data System (NPDS) and the FDA Adverse Event Reporting System (FAERS) database call frequency and type [16,18,19,20]. Furthermore, between January 2020 and July 2021, the American Association of Poison Control Centers (AAPCC) [17] received thousands of accidental exposure reports of children age 12 and younger. Certain impurities may pose acute human health risks at sufficient doses. Methanol exposure at sufficient levels, for example, may result in metabolic acidosis, headaches, dizziness, the inability to coordinate muscle movement (ataxia), and, in extreme cases, blindness, coma, seizures, and death via ingestion or dermal routes [21,22]. Further, ingesting impurities at certain levels, including benzene, acetal, acetone, ethyl acetate, 2-butanol, isobutanol, 1-butanol, isoamyl alcohol, and amyl alcohol, may result in nausea, vomiting, diarrhea, staggering gait, drowsiness, central nervous system (CNS) depression, loss of consciousness, altered mental status, and/or abdominal pain. Dermal exposure to these same impurities at sufficient levels may result in irritation, redness, burning sensation, blistering, inflammation, and/or dry skin [22,23,24,25,26,27,28,29,30,31]. Additionally, benzene and acetaldehyde specifically are listed as “carcinogenic to humans” and “possibly carcinogenic to humans”, respectively, by the International Agency for Research on Cancer (IARC) [32,33]. Whether the levels potentially present in hand sanitizers and their frequency of use would pose a risk for cancer to consumers, however, remains unknown.

This study’s primary objectives were to: (a) perform a survey of hand sanitizers marketed to children available for purchase in the U.S. during the COVID-19 global pandemic, including information on the prevalence of certain inclusion criteria that may increase attractiveness of these products to children; (b) quantify the alcohol (ethanol) percentage in the final formulation; and (c) determine if any impurities, such as methanol and other organic impurities, were present in a selection of hand sanitizers marketed to children using a modified version of FDA analytical methods.

## 2. Materials and Methods

### 2.1. Survey of Hand Sanitizers Marketed to Children

A survey of hand sanitizers marketed to children available for purchase in the U.S. during the COVID-19 pandemic was performed between January and April 2021. Qualitative data were collected for hand sanitizers marketed to children available for purchase in ‘brick and mortar’ retail stores located in California, Colorado, and Hawaii, or available online for purchase from retailers’ websites or solely online marketplaces. Store types targeted for the market survey, either online or in-person, fell into distinct store categories, including grocery stores, full-line discount stores, pharmacy/drug stores, specialty stores, and department stores. In some cases, retailers fit into more than one category, having both ‘brick and mortar’ locations and functioning as an online retailer. Store type categories and definitions are outlined in Table 1.

Hand sanitizers marketed to children were defined as having one or more of the following characteristics: (a) labeling or packaging with cartoon characters, children’s television shows, movies, toy images or references, or other pop-culture references well-known by children; (b) bright, colorful, or glittery packaging and/or liquid/gel; and/or (c) scented gel or liquid, especially a food or candy scent. Descriptive information was collected for each product, including: (a) store of purchase; (b) manufacturer name; (c) country of manufacture; (d) lot number; (e) active ingredient type and percentage; (f) scent; and (g) whether the product fit inclusion criteria.

### 2.2. Quantification of Impurities in a Selection of Hand Sanitizers Marketed to Children

A subset of 31 hand sanitizers marketed to children were purchased from ‘brick and mortar’ or online retailers and analyzed for methanol and other organic impurities listed in the FDA guidance (Table 2). One bottle of ABHS from an established and well-known manufacturer was included as a control comparison, resulting in a total of 32 samples analyzed for impurities. The control product was colorless, odorless, and did not have any packaging characteristics (e.g., colorful packaging, scents, etc.) as defined in the market survey methods. The test products were selected based on the following criteria: (a) all contained alcohol [either denatured alcohol, unspecified alcohol, or ethanol (ethyl alcohol)]; and (b) represented a variety of different manufacturers or distributors in order to characterize the maximum number of entities. Two of each hand sanitizer product were purchased at the same store, and one set was shipped to the laboratory for analysis. The other set was retained in case reanalysis was needed.

### 2.3. Sample Analysis

The FDA developed an analytical method to assess the quality of finished hand sanitizer products entitled “Direct Injection Gas Chromatography Mass Spectrometry (GC/MS) Method for the Detection of Listed Impurities in Hand Sanitizers” in August 2020 [10]. This procedure was intended to evaluate products formulated with either ethanol or isopropyl alcohol (also called isopropanol or 2-propanol) as the labeled active ingredient and 12 potential impurities (Table S1). This method was modified to include mass spectrum matching for each analyte of interest to those contained within the NIST/EPA/NIH Mass Spectral Library [34]. Each compound of interest was also quantified using a five-point calibration curve produced by the analysis of analytical standards for each analyte. These changes greatly improved identifying and quantifying compounds over the proposed method. Quality Assurance and Quality Control (QA/QC) requirements were also modified to adhere to Good Laboratory Practices (GLP). GLP regulations are intended to assure data quality and data integrity. Analytical equipment, procedures, QA/QC, and reporting requirements are listed below.

All samples were analyzed on an Agilent 5975C, Quadrupole GC/MS equipped with an Agilent 7890A Gas Chromatography and Gerstel MultiPurpose MPS2 AutoSampler with an Agilent DB-624 Capillary GC Column, 30 m × 0.25 mm × 1.4 μm. The reference standards (purity, >95%) were purchased from Sigma Aldrich, St. Louis, MI, USA, and the diluent acetonitrile was LCMS/HPLC grade, 99.9% (purchased from J.T. Baker in Phillipsburg, NJ, USA). To determine the hand sanitizer sample’s density, a 200 µL sample was weighed with an analytical balance to the nearest tenth of a milligram (±0.1 mg). Reporting units were g mL^−1^. To measure ethanol content, samples were prepared by transferring 100 µL via a micropipette to a 10 mL volumetric flask containing approximately 8 mL of acetonitrile. Samples were diluted to volume with acetonitrile and mixed. Next, 100 μL of the acetonitrile mixture was transferred to a 2 mL GC vial containing 1 mL of the internal standard (400 ng, toluene) in acetonitrile. To measure impurities, each sample was prepared by transferring 100 µL via a micropipette to a 2 mL GC vial containing 1 mL of the internal standard (400 ng, toluene) in acetonitrile. Peak assignment for each analyte is based on matching the chromatographic retention time to the reference standard (Figure S1). Peak identification is determined by mass spectral matching to the reference mass spectrum contained in NIST’s 2011 library (NIST 2021). To quantify active ingredients and impurities, a five-point calibration curve (R^2^ > 0.985) was used at concentrations bracketing the compound of interest. The curves for each compound were produced by plotting the area of each Quantitation Ion (Table S1) for the internal standard (400 ng, toluene) and reference standards. Reporting units were µg mL^−1^ (ppm), µg g^−1^ (ppm), and % by weight. Quantitation software (MSD ChemStation E.02.00.493, Agilent Technologies, Inc., Santa Clara, CA, USA) was used to determine the LOQ for each compound. The limit of quantification (LOQ) for each impurity is shown in Table 2. The hand sanitizer active ingredients and impurities, chromatogram peak assignments, LOQ, and calibration range are summarized in Table S2.

A blank and a duplicate sample were analyzed with each batch of 20 or fewer hand sanitizer samples. For samples that were measured at concentrations above the FDA interim limit, a duplicate analysis was performed to verify the results. Additionally, some products were chosen at random and a duplicate analysis was performed as an additional QC step. A total of 12 samples were tested in duplicate to confirm impurity concentrations and alcohol content. The target relative % deference (RPD) for the duplicate analysis was <30%.

### 2.4. Data Analysis

Contaminant concentrations reported as non-detect (ND) during sample analysis were calculated as one half the reported LOQ (i.e., LOQ/2) when graphing data and comparing concentrations to FDA interim limits.

Statistical analyses were conducted using SigmaPlot (SPW 14.0; Systat Software, Inc., San Jose, CA, USA). Briefly, normality was tested using Shapiro–Wilk test. For data that were normally distributed (parametric), Student’s *t*-test (two groups) or One-Way ANOVA (three groups) were used to deduce statistical differences. For data that were not normally distributed (non-parametric), Mann–Whitney Rank Sum test (two groups) or Kruskal–Wallis or One-Way ANOVA on Ranks (three groups) were used to deduce statistical differences. Additionally, paired *t*-test (parametric) or Wilcoxon Signed Rank test (non-parametric) were used to assess statistical differences between original testing and upon repeated testing (or duplicate). Specifically, statistical differences between impurity concentrations in U.S. and non-U.S. hand sanitizers, gel and liquid products, active ingredient type (ethyl alcohol, denatured alcohol, or ’unspecified alcohol’), scented or non-scented products, as well as between colored and non-colored hand sanitizers were examined (Figures S2–S7). In all cases, *p*-value < 0.05 was considered significant. The statistical tests for all comparisons are provided in Table S2.

## 3. Results

### 3.1. Survey of Hand Sanitizers Marketed to Children Available during the COVID-19 Pandemic

A total of 139 hand sanitizers potentially marketed to children were identified in the survey, 74 of which were available from brick and mortar stores, and 65 of which were available from online retailers. The majority of these products (*n* = 120, 87%) were available in full line discount stores and grocery stores. The hand sanitizers marketed to children were manufactured in the U.S. (*n* = 51, 37%), China (*n* = 85, 61%), Turkey (*n* = 1, 0.72%), or South Korea (*n* = 2, 1.44%). The hand sanitizers contained a variety of active ingredients, including ethyl alcohol (ethanol), ‘denatured alcohol’, ‘unspecified alcohol’, chloroxylenol, or benzalkonium chloride. Ethyl alcohol was the most common active ingredient, accounting for 64% (*n* = 89), and the labeled percentage of active ingredient ranged between 62 to 80%. Gel was the most common hand sanitizer type, accounting for 78% (*n* = 108) of the products, and the remainder were spray (*n* = 19), liquid (*n* = 8), or foam (*n* = 4). Almost half the hand sanitizers (*n* = 68, 49%) had labeling or packaging featuring cartoon characters, children’s television shows, movies, toy references, or other well-known pop-culture references that children would likely recognize (e.g., Disney characters). Most of the products (*n* = 105, 75%) were scented, and the rest were unscented (*n* = 33, 25%). Over 50 different scents were identified (e.g., Berry; Bubble Gum; and Strawberry Pound Cake). Three of the samples had packaging reminiscent of food packaging, such as a baby food type pouch with a screw top or honey bear shaped bottle. Several of the hand sanitizers had explicit instructions on the label regarding their use on or by children. These instructions read: (a) “Not for children under 3 years. If swallowed, get medical help or contact a Poison Control Center right away”; (b) “Keep out of reach of children”; or (c) “For children under 8, use under adult supervision.”

### 3.2. Selection of Hand Sanitizers Marketed to Children Purchased for Impurity Analysis and Ethanol Content

Table 3 provides a summary of the subset of hand sanitizers marketed to children sampled (*n* = 31), including information such as an assigned manufacturer unique identifier, active ingredient type and percentage, scent, color, and packaging information. The products selected for chemical analysis had similar attributes as those noted for all surveyed hand sanitizers. All the products analyzed in this study had at least one of the inclusion criteria that made the product potentially attractive to children, such as overt children’s packaging with cartoon characters, food or candy scents, and/or colored gel.

**Figure 2:**
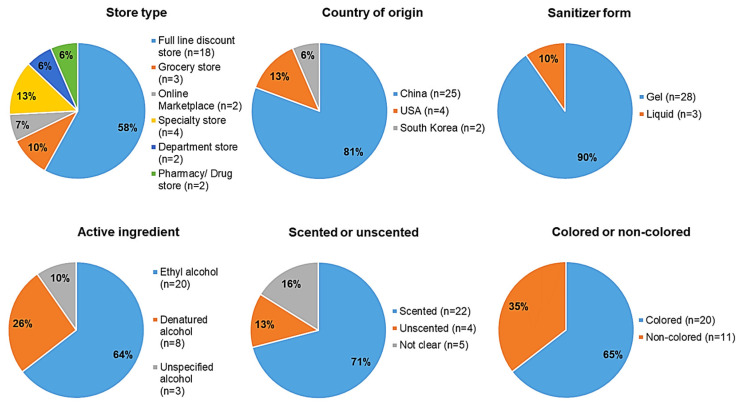
Key attributes of Hand Sanitizers Marketed to Children Selected for Testing.

The key attributes of the hand sanitizer subset selected for sampling are summarized in Figure 2. The selected hand sanitizers were manufactured in China (*n* = 25, 83%), U.S. (*n* = 4, 13%), or South Korea (*n* = 2, 6%), and represented 21 separate manufacturers. The products were labeled as containing at least one of these active ingredients: (1) ethyl alcohol (*n* = 20, 65%); (2) denatured alcohol (*n* = 8, 26%); or (3) unspecified alcohol (*n* = 3, 9%). Denatured alcohol and alcohol, however, are other names for ethyl alcohol. The percentage of labeled active ingredient ranged from 62 to 80%. The products were purchased at a wide variety of store types and locations, including at full line discount stores (*n* = 18), department stores (*n* = 2), grocery stores (*n* = 3), an online marketplace (*n* = 2), pharmacies/drug stores (*n* = 2), or specialty stores (*n* = 4). The majority of the products were in gel form (*n* = 28, 90%). The products tested primarily had a fruit scent or fruit in combination with another scent (e.g., cosmic cherry; vanilla bean + coconut + sugared musk) (*n*= 15, 48%), followed by a dessert or candy scent (e.g., cotton candy; creamy cappuccino) (*n* = 5, 16%), and the remaining samples had floral or herbal scents, or were labeled as unscented or unspecified (*n* = 11, 35%). Most products had a colored gel or liquid (*n* = 20, 64%). Only three of the products had no discernable packaging that would appeal to children specifically, whereas the rest of the products had cartoon characters or children’s TV characters on the packaging or holders attached to the bottles themselves, food or candy images, or was a well-known children’s brand.

### 3.3. Impurities and Ethanol Content Measured in a Selection of Hand Sanitizers Marketed to Children

**Figure 3:**
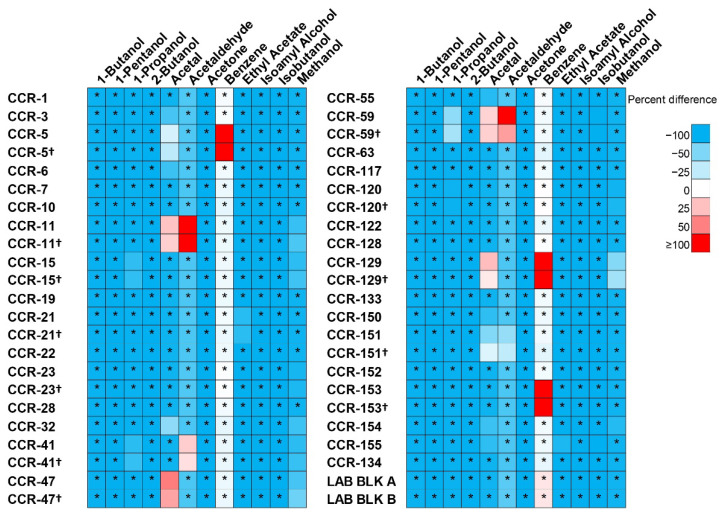
Impurity concentrations (expressed as percent difference relative to FDA specifications). Duplicate samples noted with † (expressed as percent difference relative to FDA specifications). Cells denoted with asterisks (*) refer to instances when impurity concentrations were equivalent to one half LOQ.

Table 4 lists the concentrations of organic impurities and percentage of active ingredients (%) in the children’s hand sanitizer analyzed in this study. No impurities were detected above LOQ for the control sample. Several compounds, such as 1-butanol, 1-pentanol, 2-butanol, acetone, and isoamyl alcohol, were not detected at levels above the LOQ in all children’s samples tested. The remaining impurities were measured in some of the products at the following concentrations: 1-propanol (*n* = 4, BLOQ-651 µg/g); acetal (*n* = 13, BLOQ-76 µg/g); acetaldehyde (*n* = 4, BLOQ-134 µg/g); benzene (*n* = 3, BLOQ-9.14 µg/g); ethyl acetate (*n* = 2, BLOQ-363 µg/g); and methanol (*n* = 9, BLOQ-400 µg/g). Of all the impurities tested, the most commonly measured were acetal (42% of samples) and methanol (29% of samples). Of the 31 children’s hand sanitizers, seven had concentrations of impurities above the FDA interim limits. Specifically, four bottles (CCR-11, CCR-47, CCR-59, and CCR-129) had levels of acetal higher than the FDA interim limit of 50 ppm (µg/g). With respect to benzene, all three bottles (CCR-5, CCR-129, CCR-153) containing measured concentrations were in exceedance of the FDA interim limit of 2 ppm (µg/g). Further, three bottles (CCR-11, CCR-41, CCR-59) had levels of acetaldehyde higher than the FDA interim limit of 50 ppm (µg/g). Figure 3 details a comparison between impurity concentrations measured in hand sanitizers and FDA interim limits.

To better understand differences between impurity concentrations measured in the children’s products and various characteristics, several statistical comparisons were made for U.S. and non-U.S. products, clear and non-clear liquid, and original and duplicate samples (Figures S2–S7). Samples collected from hand sanitizers manufactured in the U.S. had higher 1-propanol and isobutanol concentrations when compared to non-U.S. brand hand sanitizers. Additionally, acetal and methanol concentrations were higher for non-U.S. brands when compared to U.S. brands. However, no impurity concentrations were significantly different when comparing U.S. to non-U.S, except for methanol, which was detected in concentrations significantly higher in non-U.S. manufactured hand sanitizers. Further, samples collected from hand sanitizers with non-colored liquid reported higher 1-propanol, isobutanol, and methanol concentrations when compared to hand sanitizers with colored liquid. Additionally, acetal, benzene, and ethyl acetate concentrations were higher for colored liquid hand sanitizers compared to non-colored hand sanitizers. All impurity concentrations, however, were not significantly different when comparing non-colored and colored hand sanitizer liquid. With the exception of methanol, all impurities measured were not statistically different between duplicate samples. For sample CCR-120, methanol had significantly higher concentrations in the duplicate compared to the original dataset. No statistically significant differences in other analyte concentrations were seen in any of the 12 hand sanitizer bottles that were tested in duplicate (Figure S7).

### 3.4. Ethanol Content Measured in a Selection of Hand Sanitizers Marketed to Children

The measured alcohol content ranged from 52% to 98% in all tested hand sanitizers marketed to children. The alcohol percentage reported on the bottle labels, however, ranged from 62% to 80%. Figure S8 shows a comparison between labeled and measured alcohol concentrations for each product. Overall, when comparing the measured and labeled alcohol ranges, the measured concentrations in the bottles ranged from 31% lower than the labeled concentration to up to 28% higher than the labeled concentration, depending on the product. Almost one third (*n* = 10) of the bottles tested had alcohol concentrations lower than the required 60% needed for germicidal properties, as recommended by FDA and CDC [3,22].

## 4. Discussion

The survey identified numerous products potentially attractive to children available for purchase in the U.S. The majority of the products contained fragrances and coloring additives that might increase their appeal, despite FDA recommending against these additives because of exposure risk in children. Further, we found that products continued to be packaged in containers resembling food or drink containers, despite FDA alerts to similar products because of their possible accidental ingestion risk by children [12]. Hence, understanding the possible health risks associated with incidental ingestion and dermal absorption – especially by children – of hand sanitizer impurities is needed not only to effectively characterize potential future health risks, but also to help successfully manage them. Tse et al. (2021) reported that, based on the toxicology of common impurities found in technical-grade ethanol, the majority pose a low risk during normal use [35]. However, acetaldehyde and benzene, in particular, may pose a risk especially to susceptible populations like children, and potential risk from exposure to several impurities is still uncertain. Further, certain formulation additives (such as water or gelling agents) may increase the risk of dermal exposure and should be studied further [35]. In a concurrent study by Kozal et al. presented at the Society of Toxicology (SOT), a screening level risk assessment was performed to estimate systemic exposure doses (SEDs) that may result from repeated dermal exposure, as well as incidental ingestion of hand sanitizers, and were compared to health-based guidance values and toxicity thresholds [36].

Numerous product recalls and alerts were issued by FDA during the COVID-19 pandemic due to methanol contamination. The full list of recalled products and associated warning letters detailing the methanol concentrations are available on FDA website [12]. As an example, methanol concentrations in hand sanitizers manufactured by 4E Global SAPI de CV were reported to range from 65% to 74% methanol *v*/*v* [37]. Although several alerts were reported for contamination with benzene, acetaldehyde, or acetal, detailed warning letters specifying concentrations of these contaminants were not available on FDA website. In one alert, a product was found to contain 6 ppm benzene [38]. Therefore, it is unclear if the concentrations reported in our study are similar to the concentrations found by FDA, as this information is not publicly available. One of the products (CCR-129) with levels of acetal that exceeded FDA interim limits was listed on the ‘FDA’s Do-Not-Use List’ in April 2022 due to the product being manufactured “at the same facility that produced benzene contaminated product” [12]. Additionally, numerous products from the same distributor as several products tested in this study were also included on the FDA list because they were manufactured at the same facilities in which a product was found to have methanol and benzene contamination. Another product tested in this study was recommended for recall, and was added to an import alert in March 2022.

Several other studies have reported impurity concentrations measured in ABHSs. In an unpublished study, 260 unique batches of hand sanitizer were analyzed and 44 batches (17%) contained benzene at 0.1 ppm or above, while 21 batches (8%) contained benzene at 2 ppm or above [39]. The highest benzene concentration detected was 16.1 ppm, over eight times the FDA interim limit of 2 ppm [39]. Of the 21 batches that contained >2 ppm benzene, one batch reported 8680 ppm methanol and 147 ppm acetaldehyde, which are fourteen and three times the FDA interim limit, respectively. Another batch contained 709 ppm methanol [39]. In another study, 42 liquid and gel hand sanitizers were analyzed for nine different impurities, and 11 of the samples were non-compliant with interim Health Canada guidelines [11]. The authors noted that these samples primarily contained acetaldehyde at levels above the Canadian interim guidance of NMT 75 ppm total acetaldehyde and acetal [40]. In a study conducted in the U.S., 51 samples from bulk refillable hand sanitizer dispensers at community settings, such as restaurants, malls, and fitness centers, across the U.S., and 40 samples from a single school district in South Carolina were analyzed for methanol, benzene, acetaldehyde, and acetal [41]. Only one sample from the school district was positive for acetal (511 ppm) at levels above the FDA interim limit, while 35.29% (18/51), 33.33% (17/51), and 5.88% (3/51) of the community-acquired samples had acetal, acetaldehyde, and methanol concentrations, respectively, above the FDA’s interim limits. Benzene was not detected in any of the 91 samples [41]. Further, in a study conducted in Malaysia, of the 121 samples purchased from retail locations, 7.4% contained methanol above the LOD of 4.4% (*v*/*v*), while 18.8% of the 265 samples collected from freely deployed public dispensers contained methanol above the LOD [42]. A recent study by Pal et al. (2022) tested 200 hand sanitizers for concentrations of benzene, toluene, and styrene [43]. Similar to our study, the authors found that a selection of products (*n* = 10) exceeded FDA interim limits for benzene (>2 ppm). It was also noted that products also contained toluene (25%) and styrene (32%); however, these compounds have no FDA limits. Similar to our study, the hand sanitizers purchased were primarily manufactured in the U.S. or China; however, they also identified additional countries (such as India, Mexico, and United Arab Emirates) that were not represented in our study. Therefore, it is possible that there are additional impurities present in hand sanitizers that were not quantified in our study, but their presence may differ based on the country of manufacture. Further, the authors performed an exposure assessment and determined that the benzene exposures would “increase the EPA’s benchmark for the *de minimus* cancer risk” in children, teenagers, and adults [43].

Certain ABHSs identified in this study may possibly be less effective at preventing SARS-CoV-2 transmission from hand surfaces due to lower ethanol content than current regulatory standards; however, more information would be needed to verify that hypothesis. Some studies have reported on ethanol content in hand sanitizer products. For example, Tse et al. (2021) [11] reported ethanol concentrations between 63% and 90% *v*/*v*, with one sample having ethanol content below 60%. Another study reported ethanol concentrations between 16.21% and 87.33% *v*/*v*, with a total of 33.62% of the samples collected (39/116) containing under 60% ethanol [41].

The current study was not without limitations. One limitation included the GC/MS analysis sensitivity for detecting benzene. The GC/MS analytical method utilized in this study resulted in an LOQ of 0.44 µg/mL for benzene. The determined LOQ met the FDA’s concentration ranges reported in its published method entitled “Direct Injection Gas Chromatography Mass Spectrometry (GC/MS) Method for the Detection of Listed Impurities in Hand Sanitizers” (0.044–2.19 µg/mL). In terms of sensitivity, however, the determined LOQ was one order of magnitude larger than the lowest range that FDA reported. Overall, the determined LOQ met method specifications; however, when converting two reported benzene non-detects from one hand sanitizer marketed to children (CCR-120 and CCR-120 duplicate) utilizing the measured sample density (0.9145 g/mL) to a µg/g value in order to compare to the FDA interim limit, the calculated concentration appeared to be above the FDA interim limit. All other benzene non-detects, when converted to µg/g utilizing hand sanitizer densities measured between 0.8345–0.8865 g/mL, were below the FDA interim limit. An additional study limitation was that our dataset provides impurity levels in the final formulation, which contain other ingredients such as fragrances, dyes, water, and other compounds, and not in the raw ethanol. The concentrations therefore may be higher in the raw ethanol than reported in this study, and additional exceedances cannot be ruled out. Further, the impurities possibly could have come from ingredients other than the raw ethanol.

## 5. Conclusions

Given increased ABHS consumer use during the COVID-19 pandemic and the potential ingestion risk by children, characterization of the availability of ABHSs that may be attractive to children proved vital. Further, due to increased reports of incidental ingestion and potential contamination of these products with impurities and ineffective ethanol levels, identification and quantification of potential health hazards to the public, especially to children, who represent a unique at-risk population, was performed.

As this study shows, numerous ABHSs with labeling or packaging characteristics that may increase their attractiveness to children are available for purchase in the U.S. Some products were packaged in containers resembling food, which is concerning because of the possibility of accidental ingestion. In our impurity analysis of a subset of available ABHSs marketed to children, the majority of the products did contain measurable concentrations of one or more organic impurities at levels above the respective LOQs. However, only some of the products exceeded FDA interim limits for several organic impurities, such as acetaldehyde, benzene, and acetal. All products were analyzed in their final formulation; however, the FDA interim limits were developed to address the acceptable impurity levels in the raw ethanol used to manufacture these products. Whether the impurity levels seen in this study would be higher in the raw ethanol remains unclear. While we observed exceedances, it is unclear if the impurities are present at concentrations that would possibly pose a health risk to consumers—especially children—should dermal absorption or accidental ingestion occur. Given the potential for exposure to children, however, and the reported accidental poisoning increase in children following the start of the COVID-19 pandemic, this issue is concerning, and should be further evaluated.

Future studies on the potential source(s) of these impurities in ethanol and/or other ingredients used in hand sanitizer products are thus needed. Understanding if potential contamination was a direct result of new manufacturers and improper manufacturing procedures during the COVID-19 pandemic would be helpful, as would knowing if the presence of such impurities will linger even after the FDA interim limits are no longer applicable and the ethanol shortage has diminished. Additional assessments should be made to determine whether the criteria examined in this study, such as packaging, gel coloring, and scent, encourage ABHS attraction, use, and potential ingestion. We can reasonably assume that future events may once again increase ABHS demand, and result in supply shortages.

## Figures and Tables

**Table 1:** Store Type Categories and Definitions.

Store Type		Definition
Type 1	Brick and mortar	Store with physical location that is visited in person
	Online retailer	Store with ’online’ presence that is visited from any device with internet access
Type 2	Grocery store	Store that sells primarily food for consumption
	Full line discount store	Store that sells various types of products, including groceries
	Pharmacy/drug store	Store that sells prescription drugs, OTC drugs, as well as medical supplies and groceries
	Distillery	A place or establishment that distills liquor
	Specialty store	Store that carries a deep assortment of brands, styles, or models within a relatively narrow category of goods
	Department store	Retail establishment offering a wide range of consumer goods in different areas of the store; each area (‘department’) specializes in a product category

**Table 2:** List of FDA Interim Limits and Limits of Detection/Limits of Quantification (LODs/LOQs) for Impurities Assessed in Hand Sanitizers Marketed to Children.

Chemical Name	CAS Number	Interim Limit Listed in FDA Guidance (ppm) ^a,b^	LOD/LOQ (µg/g) ^c^
1-Propanol	71-23-8	NMT 1000	68.3
Methanol	67-56-1	NMT 630	67.2
Benzene	71-43-2	NMT 2	3.74
Acetaldehyde	75-07-0	NMT 50	30.0
Acetal	105-57-7	NMT 50	5.30
Acetone	67-64-1	NMT 4400	67.2
Ethyl Acetate	141-78-6	NMT 2200	76.6
2-Butanol	78-92-2	NMT 6200	68.7
Isobutanol	78-83-1	NMT 21,700	68.3
1-Butanol	71-36-3	NMT 1000	68.9
Isoamyl alcohol	123-51-3	NMT 4100	68.8
Amyl alcohol	71-41-0	NMT 4100	68.9

^a^ NMT = Not more than. ^b^ Methanol, benzene, and acetaldehyde have specific individual limits. The sum of all other impurities should not exceed 300 ppm. If the sum of all other impurities is >300 ppm, then each impurity in this category must meet the individual limit specified above. ^c^ Based on sample volume of 0.1 mL, density 0.850 g/mL.

**Table 3:** Selection of Hand Sanitizers Marketed to Children Tested for Impurities.

Sample Ref. No.	Manufacturer Identification Code	Country of Manufacture	Active Ingredient	Labeled Active Ingredient (%)	Store Type	Labeled Scent	Scent Category	Sanitizer Form	Gel/Liquid Color	Packaging Category
CCR-1	Manufacturer A	China	Ethyl Alcohol	70	Full line discount store	Unscented	No scent	Gel	Non-colored	Cartoon Character
CCR-3	Manufacturer B	China	Ethyl Alcohol	62	Full line discount store	Choco Orange	Fruit/Candy	Gel	Orange	Candy Image
CCR-5	Manufacturer C	China	Ethyl Alcohol	63	Full line discount store	Cotton Candy	Candy	Gel	Purple	Cartoon Character; Candy Image
CCR-6	Manufacturer C	China	Ethyl Alcohol	63	Full line discount store	Berry	Fruit	Gel	Pink	Cartoon Character; Food Image
CCR-7	Manufacturer D	China	Denatured alcohol	70	Full line discount store	Cotton Candy	Candy	Gel	Pink	Cartoon Character; Glitter
CCR-10	Manufacturer E	China	Ethyl Alcohol	70	Full line discount store	Ocean Breeze	Un-categorized	Gel	Non-colored	Cartoon Character
CCR-11	Manufacturer F	China	Ethyl Alcohol	75	Full line discount store	Strawberry	Fruit	Gel	Pink	Food Image
CCR-15	Manufacturer F	China	Ethyl Alcohol	75	Full line discount store	Vanilla Sugar Cookie	Dessert	Gel	Green	Food Image
CCR-19	Manufacturer G	China	Ethyl Alcohol	75	Full line discount store	Unscented	No scent	Gel	Non-colored	Cartoon Character (outer cover)
CCR-21	Manufacturer H	China	Unspecified Alcohol	65	Full line discount store	Cotton Candy	Candy	Gel	Pink	Cartoon Character (outer cover); Candy Image
CCR-22	Manufacturer I	China	Denatured alcohol	75	Full line discount store	Citrus	Fruit	Gel	Non-colored	Cartoon Character
CCR-23	Manufacturer J	China	Denatured alcohol	62	Pharmacy/Drug Store	Berry Lollipop	Fruit/Candy	Gel	Pink	Candy Image
CCR-28	Manufacturer J	China	Denatured alcohol	62	Pharmacy/Drug Store	Fruit Punch	Fruit	Gel	Pink	Children’s TV Character
CCR-32	Manufacturer D	China	Denatured alcohol	70	Grocery Store	Grape	Fruit	Gel	Purple	Cartoon Character
CCR-41	Manufacturer D	China	Denatured alcohol	70	Grocery Store	Raspberry	Fruit	Liquid	Pink	Cartoon Character
CCR-47	Manufacturer D	China	Denatured alcohol	70	Grocery Store	Strawberry	Fruit	Liquid	Pink	Children’s TV Character
CCR-55	Manufacturer K	China	Ethyl Alcohol	68	Full line discount store	Unscented	No scent	Liquid	Non-colored	Cartoon Character (outer packaging)
CCR-59	Manufacturer L	USA	Ethyl Alcohol	80	Full line discount store	Lavender	Floral	Gel	Non-colored	Honey bear shaped packaging
CCR-63	Manufacturer M	USA	Ethyl Alcohol	68	Full line discount store	Strawberry Pound Cake	Dessert	Gel	Non-colored with blue beads	None
CCR-117	Manufacturer N	USA	Ethyl Alcohol	75	Online retailer	Unscented	No scent	Gel	Blue	Kid’s Brand
CCR-120	Manufacturer O	China	Ethyl Alcohol	63	Online retailer	Unscented	No scent	Gel	Non-colored	Cartoon Character
CCR-122	Manufacturer H	China	Denatured alcohol	66	Pharmacy/Drug Store	Melon	Fruit	Gel	Pink	Food Image
CCR-128	Manufacturer K	China	Ethyl Alcohol	68	Specialty Store	Unscented	No scent	Gel	Non-colored	Cartoon Character (outer packaging)
CCR-129	Manufacturer P	South Korea	Ethyl Alcohol	70	Department Store	Candy Apple	Fruit/Candy	Gel	Non-colored	Cartoon Character; Candy Image; Pouch packaging
CCR-133	Manufacturer B	China	Unspecified Alcohol	62	Full line discount store	Berries & Cream	Dessert	Gel	Pink	Food Image
CCR-134 (Control)	Manufacturer Q	USA	Ethyl Alcohol	70	Full line discount store	Unscented	No scent	Gel	Non-colored	None
CCR-150	Manufacturer P	South Korea	Ethyl Alcohol	70	Department Store	Mixed Berry	Fruit	Gel	Non-colored	Pouch packaging
CCR-151	Manufacturer R	China	Ethyl Alcohol	75	Pharmacy/Drug Store	Watermelon	Fruit	Gel	Pink	Food Image
CCR-152	Manufacturer S	China	Unspecified Alcohol	67	Specialty Store	Unscented	No scent	Gel	Orange	Holographic
CCR-153	Manufacturer K	China	Ethyl Alcohol	68	Specialty Store	Unscented	No scent	Gel	Turquoise	Cartoon Character
CCR-154	Manufacturer T	China	Ethyl Alcohol	63	Pharmacy/Drug Store	Unscented	No scent	Gel	Non-colored	Cartoon Character
CCR-155	Manufacturer U	USA	Ethyl Alcohol	62	Specialty Store	Cupcake	Dessert	Gel	Purple	None

**Table 4:** Concentrations of Impurities Measured in Selected Hand Sanitizers Marketed to Children.

	Actives Measured Concentration (%)						Impurities Measured Concentration (µg/g)							
Sample Ref. No.	Ethanol	Isopropanol	1-Butanol	1-Pentanol	1-Propanol	2-Butanol	Acetal	Acetaldehyde	Acetone	Benzene	Ethyl acetate	Isoamyl alcohol	Isobutanol	Methanol
**CCR-1**	61	-	BLOQ	BLOQ	BLOQ	BLOQ	BLOQ	BLOQ	BLOQ	BLOQ	BLOQ	BLOQ	BLOQ	BLOQ
**CCR-3**	57	-	BLOQ	BLOQ	BLOQ	BLOQ	**13.9**	BLOQ	BLOQ	BLOQ	BLOQ	BLOQ	BLOQ	BLOQ
**CCR-5**	55	-	BLOQ	BLOQ	BLOQ	BLOQ	**41.8**	BLOQ	BLOQ	**9.14**	BLOQ	BLOQ	BLOQ	BLOQ
**CCR-5** *	55	-	BLOQ	BLOQ	BLOQ	BLOQ	**37.0**	BLOQ	BLOQ	**4.85**	BLOQ	BLOQ	BLOQ	BLOQ
**CCR-6**	80	-	BLOQ	BLOQ	BLOQ	BLOQ	**11.7**	BLOQ	BLOQ	BLOQ	BLOQ	BLOQ	BLOQ	BLOQ
**CCR-7**	66	-	BLOQ	BLOQ	BLOQ	BLOQ	BLOQ	BLOQ	BLOQ	BLOQ	BLOQ	BLOQ	BLOQ	BLOQ
**CCR-10**	56	-	BLOQ	BLOQ	BLOQ	BLOQ	BLOQ	BLOQ	BLOQ	BLOQ	BLOQ	BLOQ	BLOQ	BLOQ
**CCR-11**	88	-	BLOQ	BLOQ	BLOQ	BLOQ	**61.8**	**134**	BLOQ	BLOQ	BLOQ	BLOQ	BLOQ	**134**
CCR-11 *	88	-	BLOQ	BLOQ	BLOQ	BLOQ	**59.6**	**133**	BLOQ	BLOQ	BLOQ	BLOQ	BLOQ	**187**
**CCR-15**	69	-	BLOQ	BLOQ	**189**	BLOQ	BLOQ	BLOQ	BLOQ	BLOQ	BLOQ	BLOQ	BLOQ	**109**
CCR-15 *	69	-	BLOQ	BLOQ	**192**	BLOQ	BLOQ	BLOQ	BLOQ	BLOQ	BLOQ	BLOQ	BLOQ	**156**
**CCR-19**	86	-	BLOQ	BLOQ	BLOQ	BLOQ	BLOQ	BLOQ	BLOQ	BLOQ	BLOQ	BLOQ	BLOQ	BLOQ
**CCR-21**	80	-	BLOQ	BLOQ	BLOQ	BLOQ	BLOQ	BLOQ	BLOQ	BLOQ	**340**	BLOQ	BLOQ	BLOQ
CCR-21 *	80	-	BLOQ	BLOQ	BLOQ	BLOQ	BLOQ	BLOQ	BLOQ	BLOQ	**363**	BLOQ	BLOQ	BLOQ
**CCR-22**	61	-	BLOQ	BLOQ	BLOQ	BLOQ	BLOQ	BLOQ	BLOQ	BLOQ	BLOQ	BLOQ	BLOQ	BLOQ
**CCR-23**	63	-	BLOQ	BLOQ	BLOQ	BLOQ	BLOQ	BLOQ	BLOQ	BLOQ	BLOQ	BLOQ	BLOQ	**55.3**
CCR-23 *	63	-	BLOQ	BLOQ	BLOQ	BLOQ	BLOQ	BLOQ	BLOQ	BLOQ	BLOQ	BLOQ	BLOQ	**71.5**
**CCR-28**	59	-	BLOQ	BLOQ	BLOQ	BLOQ	BLOQ	BLOQ	BLOQ	BLOQ	BLOQ	BLOQ	BLOQ	BLOQ
**CCR-32**	98	-	BLOQ	BLOQ	BLOQ	BLOQ	**28**	BLOQ	BLOQ	BLOQ	BLOQ	BLOQ	BLOQ	**150**
**CCR-41**	52	-	BLOQ	BLOQ	**166**	BLOQ	BLOQ	**60**	BLOQ	BLOQ	BLOQ	BLOQ	BLOQ	**107**
CCR-41 *	52	-	BLOQ	BLOQ	**206**	BLOQ	BLOQ	**56**	BLOQ	BLOQ	BLOQ	BLOQ	BLOQ	**138**
**CCR-47**	69	-	BLOQ	BLOQ	BLOQ	BLOQ	**76**	BLOQ	BLOQ	BLOQ	BLOQ	BLOQ	BLOQ	**174**
CCR-47 *	69	-	BLOQ	BLOQ	BLOQ	BLOQ	**68**	BLOQ	BLOQ	BLOQ	BLOQ	BLOQ	BLOQ	**273**
**CCR-55**	84	-	BLOQ	BLOQ	BLOQ	BLOQ	**8**	BLOQ	BLOQ	BLOQ	BLOQ	BLOQ	BLOQ	BLOQ
**CCR-59**	55	-	BLOQ	BLOQ	**563**	BLOQ	**59**	**104**	BLOQ	BLOQ	BLOQ	BLOQ	**175**	BLOQ
CCR-59 *	55	-	BLOQ	BLOQ	**651**	BLOQ	**59**	**69**	BLOQ	BLOQ	BLOQ	BLOQ	**221**	BLOQ
**CCR-63**	69	3	BLOQ	BLOQ	BLOQ	BLOQ	BLOQ	BLOQ	BLOQ	BLOQ	BLOQ	BLOQ	BLOQ	BLOQ
**CCR-117**	75	-	BLOQ	BLOQ	BLOQ	BLOQ	BLOQ	BLOQ	BLOQ	BLOQ	BLOQ	BLOQ	BLOQ	BLOQ
**CCR-120**	66	-	BLOQ	BLOQ	**50.1**	BLOQ	BLOQ	BLOQ	BLOQ	BLOQ	BLOQ	BLOQ	BLOQ	**37.8**
CCR-120 *	66	-	BLOQ	BLOQ	**41.4**	BLOQ	BLOQ	BLOQ	BLOQ	BLOQ	BLOQ	BLOQ	BLOQ	**66.6**
**CCR-122**	67	-	BLOQ	BLOQ	BLOQ	BLOQ	BLOQ	BLOQ	BLOQ	BLOQ	BLOQ	BLOQ	BLOQ	BLOQ
**CCR-128**	64	-	BLOQ	BLOQ	BLOQ	BLOQ	BLOQ	BLOQ	BLOQ	BLOQ	BLOQ	BLOQ	BLOQ	BLOQ
**CCR-129**	74	2	BLOQ	BLOQ	BLOQ	BLOQ	**63**	BLOQ	BLOQ	**6.94**	BLOQ	BLOQ	BLOQ	**328**
CCR-129 *	74	2	BLOQ	BLOQ	BLOQ	BLOQ	**54.4**	BLOQ	BLOQ	**4.36**	BLOQ	BLOQ	BLOQ	**400**
**CCR-133**	55	-	BLOQ	BLOQ	BLOQ	BLOQ	BLOQ	BLOQ	BLOQ	BLOQ	BLOQ	BLOQ	BLOQ	BLOQ
**CCR-150**	75	-	BLOQ	BLOQ	BLOQ	BLOQ	**9.16**	BLOQ	BLOQ	BLOQ	BLOQ	BLOQ	BLOQ	BLOQ
**CCR-151**	80	-	BLOQ	BLOQ	BLOQ	BLOQ	**25.4**	**29.6**	BLOQ	BLOQ	BLOQ	BLOQ	BLOQ	BLOQ
CCR-151 *	80	-	BLOQ	BLOQ	BLOQ	BLOQ	**43.3**	**37.5**	BLOQ	BLOQ	BLOQ	BLOQ	BLOQ	BLOQ
**CCR-152**	63	-	BLOQ	BLOQ	BLOQ	BLOQ	BLOQ	BLOQ	BLOQ	BLOQ	BLOQ	BLOQ	BLOQ	BLOQ
**CCR-153**	65	-	BLOQ	BLOQ	BLOQ	BLOQ	BLOQ	BLOQ	BLOQ	**4.67**	BLOQ	BLOQ	BLOQ	BLOQ
CCR-153 *	65	-	BLOQ	BLOQ	BLOQ	BLOQ	BLOQ	BLOQ	BLOQ	**7.91**	BLOQ	BLOQ	BLOQ	BLOQ
**CCR-154**	83	-	BLOQ	BLOQ	BLOQ	BLOQ	**11.6**	BLOQ	BLOQ	BLOQ	BLOQ	BLOQ	BLOQ	**88**
**CCR-155**	62	-	BLOQ	BLOQ	BLOQ	BLOQ	**9.3**	BLOQ	BLOQ	BLOQ	**235**	BLOQ	**119**	BLOQ
**CCR-134** **(Control)**	74	3	BLOQ	BLOQ	BLOQ	BLOQ	BLOQ	BLOQ	BLOQ	BLOQ	BLOQ	BLOQ	BLOQ	BLOQ
**Lab Blank A**	-	-	BLOQ	BLOQ	BLOQ	BLOQ	BLOQ	BLOQ	BLOQ	BLOQ	BLOQ	BLOQ	BLOQ	BLOQ
**Lab Blank B**	-	-	BLOQ	BLOQ	BLOQ	BLOQ	BLOQ	BLOQ	BLOQ	BLOQ	BLOQ	BLOQ	BLOQ	BLOQ
**Average (STD)**	68.5% (± 11%)	0.2% (± 0.7%)	34.7 (± 1.009)	34.7 (± 1.01)	75.8 (± 127.4)	34.6 (± 1.006)	18.7 (± 23.848)	26.9 (± 29.837)	33.8 (± 0.984)	2.5 (± 1.697)	57.8 (± 72.241)	34.6 (± 1.007)	44.0 (± 37.175)	78.8 (± 84.541)
**Median**	66%	0.0%	34.5	34.5	34.6	34.4	2.8	15.2	33.6	1.9	38.8	34.4	34.2	34.5
**Range**	52–98%	0–3.3%	32.8–37	32.9–37.1	32.6–651	32.8–36.9	2.5–75.6	14.3–134.1	32.1–36.1	1.8–9.1	36.6–363	32.8–37	32.6–221	32.1–400

Footnote: BLOQ: below limit of quantification; (-): not measured; (*): duplicative sample; descriptive statistics (avg, STD, median, range) did not include sample CCR-134, or Lab Blank A and B. LOQ for each analyte was calculated using sample volume 0.1 mL, and density of each sample, which ranged between 0.8115–1.0 g mL^−1^.

## Data Availability

Not applicable.

## Supplementary Materials

The following supporting information can be downloaded at: https://www.mdpi.com/article/10.3390/ijerph192114424/s1, Table S1: Hand sanitizer active ingredients and impurities, chromatogram peak assignments, LOQ and calibration range; Table S2: Statistical tests for comparing impurity concentrations in U.S. and non-U.S. products; Figure S1: Total Ion Chromatogram (TIC) of Hand Sanitizer Active Ingredients and Impurities; Figure S2: Impurity concentrations in U.S. and non-U.S. manufactured products; Figure S3: Impurity concentrations in gel and liquid products; Figure S4: Impurity concentrations in products by type of active ingredient; Figure S5: Impurity concentrations in scented or non-scented products; Figure S6: Impurity concentrations in non-colored and colored products; Figure S7: Impurity concentrations in original and duplicate samples; Figure S8: Labeled vs. measured alcohol concentrations in hand sanitizers marketed to children (expressed as a ratio fraction).
