# Supplementary material for: Evaluation of the Safety and Efficacy of Hand Sanitizer Products Marketed to Children Available during the COVID-19 Pandemic
Source: Int J Environ Res Public Health. 2022 Nov 3;19(21):14424. doi: 10.3390/ijerph192114424 (PMC9655340; doi:10.3390/ijerph192114424)
Supplement: Supplementary file 1 [file ijerph-19-14424-s001.zip › ijerph-1960371-supplementary.pdf]

International Journal of
Environmental Research and Public Health

MDPI

Supplementary Materials

Table S1. Hand sanitizer active ingredients and impurities, chromatogram peak assignments, LOQ and calibration range.

Active and Impurities	CAS number	Retention Time (Min.)	Quantitation Ion (m/z)	LOQ (μg mL^{-1})	LOQ (μg g^{-1})	Calibration range (μg/mL)	Calibration range (μg/g)
**Active Ingredient:**							
Ethanol	64-17-5	3.277	31.1	19.7	1680#	19.7 - 1970	1680 - 168000#
Isopropyl alcohol (Isopropanol)	67-63-0	4.149	45.1	19.6	1670#	19.6 - 1960	1670 - 167000#
**Impurities:**							
Acetaldehyde	75-07-0	2.158	44.0	3.53	30.0*	3.53 - 58.9	30.0 - 501*
Methanol	67-56-1	2.294	31.1	7.91	67.2*	7.91 - 791	67.2 - 6720*
Acetone	67-64-1	3.906	43.1	7.90	67.2*	7.90 - 790	67.2 - 6720*
1-Propanol	71-23-8	6.001	31.1	8.04	68.3*	8.04 - 804	68.3 - 6830*
Ethyl Acetate	141-78-6	6.614	43.1	9.02	76.6*	9.02 - 902	76.7 - 7670*
2-Butanol	78-92-2	6.743	45.1	8.08	68.7*	8.08 - 808	68.7 - 6870*
Isobutanol	78-83-1	7.056	43.1	8.03	68.3*	8.03 - 803	68.3 - 6830*
Benzene	71-43-2	7.339	78.1	0.44	3.74*	0.44 - 43.7	3.74 - 371*
1-Butanol	71-36-3	7.803	56.1	8.10	68.9*	8.10 - 810	68.9 - 6890*
(Acetal)	105-57-7	8.165	45.1	0.62	5.30*	0.62 - 62.3	5.30 - 530*
Isoamylalcohol	123-51-3	8.687	55.1	8.09	68.8*	8.09 - 809	68.8 - 6880*
Amyl Alcohol	71-41-0	8.958	42.1	8.11	68.9*	8.11 - 811	68.9- 6890*

* based on sample volume 0.1mL, density 0.850g mL-1, # based on sample volume 0.01mL, density 0.850g mL-1.

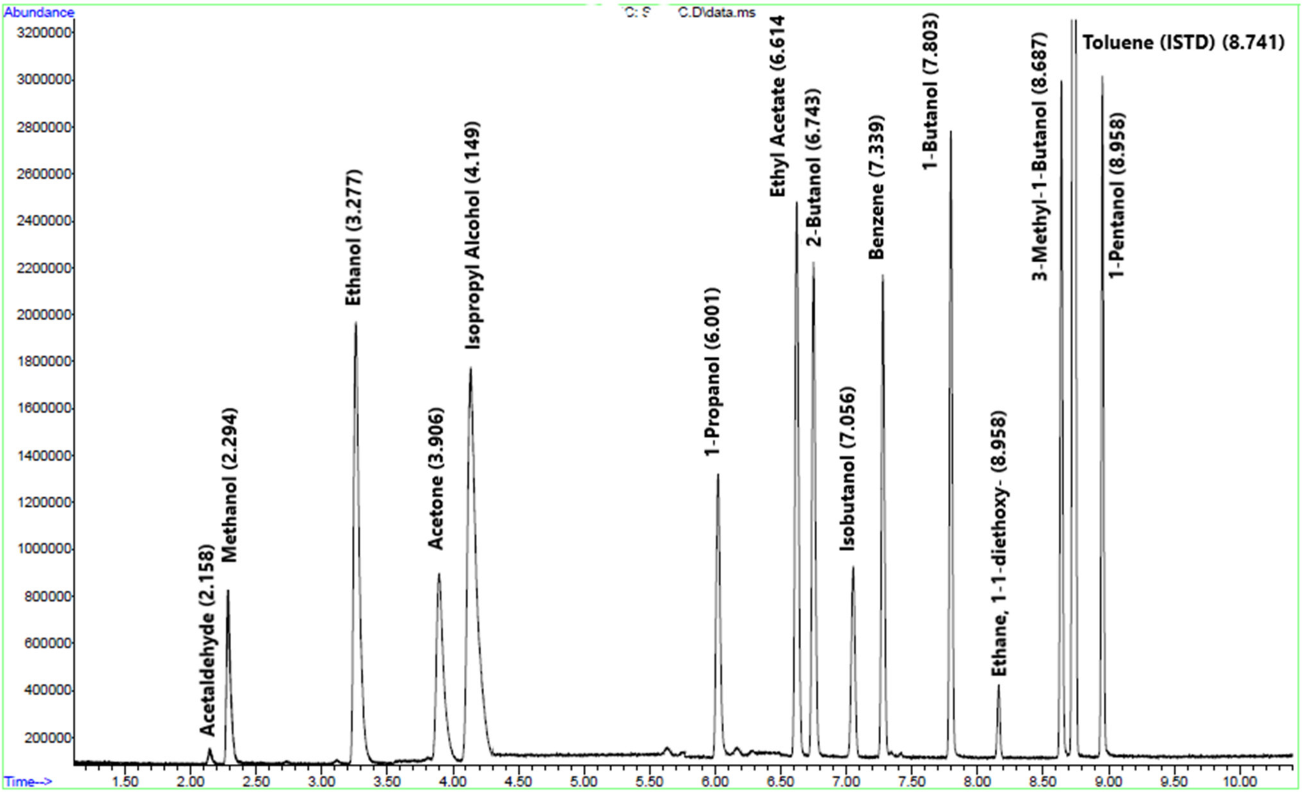

Figure S1. Total Ion Chromatogram (TIC) of Hand Sanitizer Active Ingredients and Impurities.

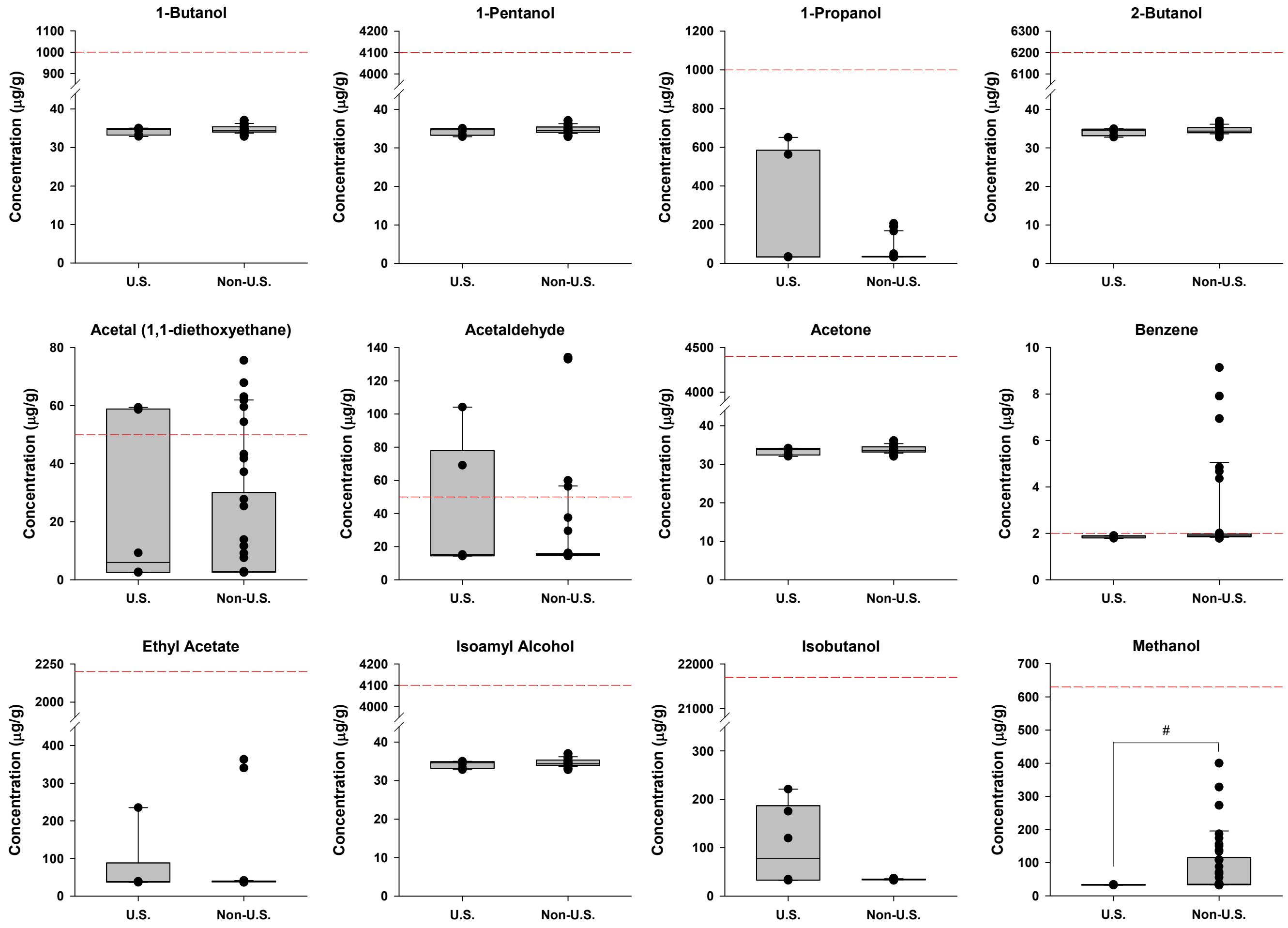

Figure S2. Impurity concentrations in U.S. and non-U.S. manufactured products. Statistical differences are indicated with a bracket and number sign. For details on statistical tests refer to Supplemental Table 2.

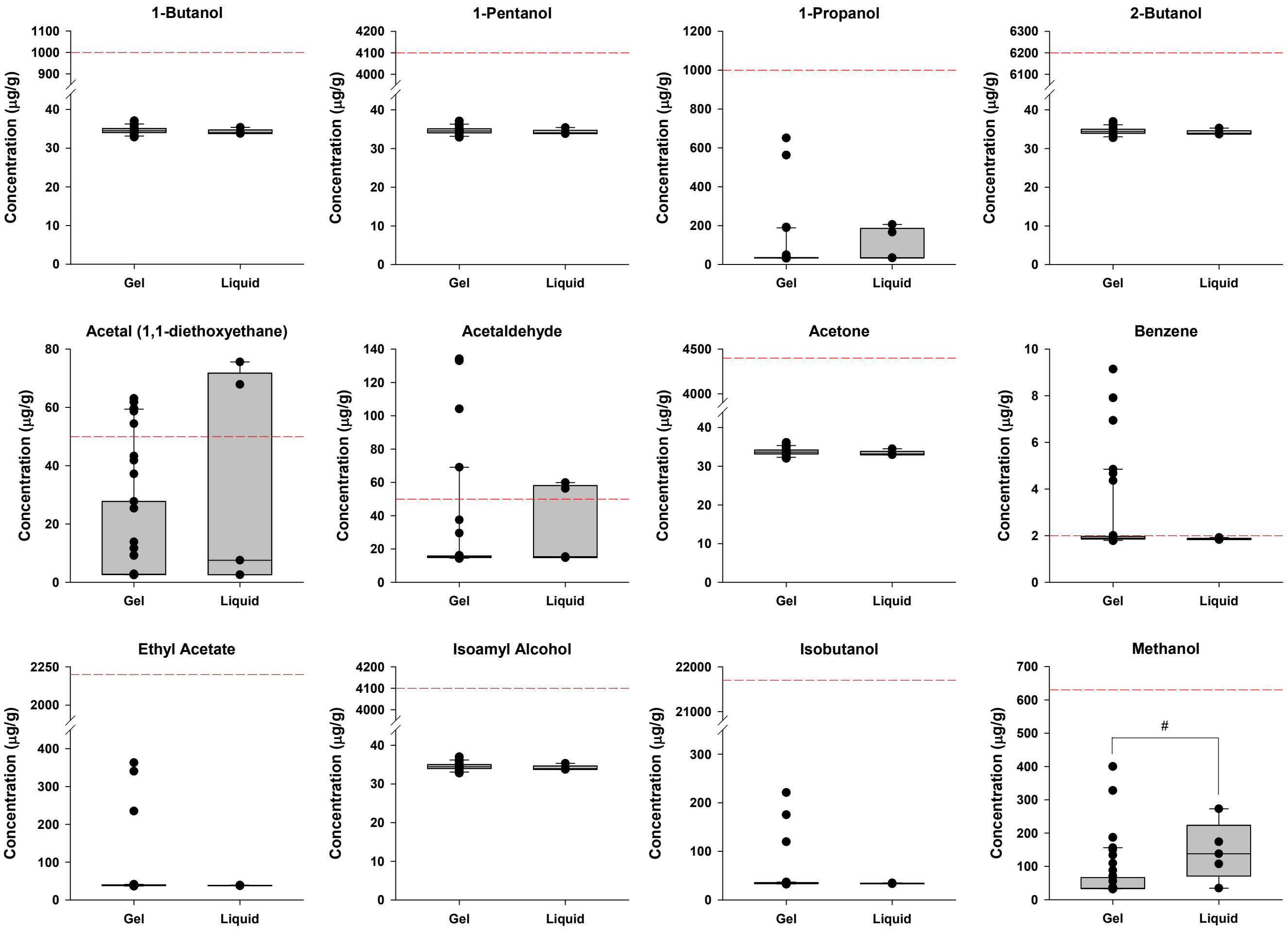

Figure S3. Impurity concentrations in gel and liquid products. Statistical differences are indicated with a bracket and number sign. For details on statistical tests refer to Supplemental Table 2.

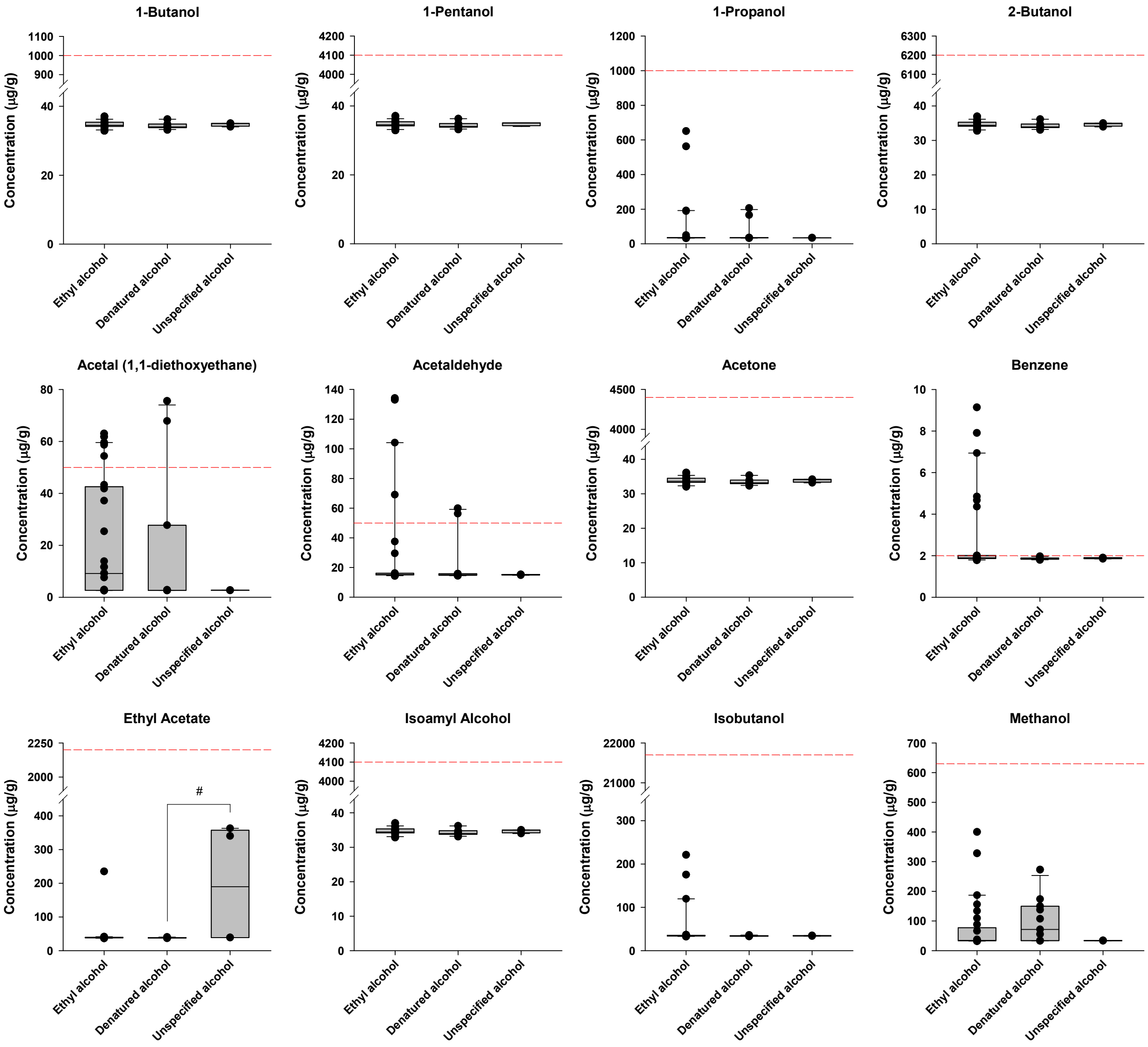

Figure S4. Impurity concentrations in products by type of active ingredient. Statistical differences are indicated with a bracket and number sign. For details on statistical tests refer to Supplemental Table 2.

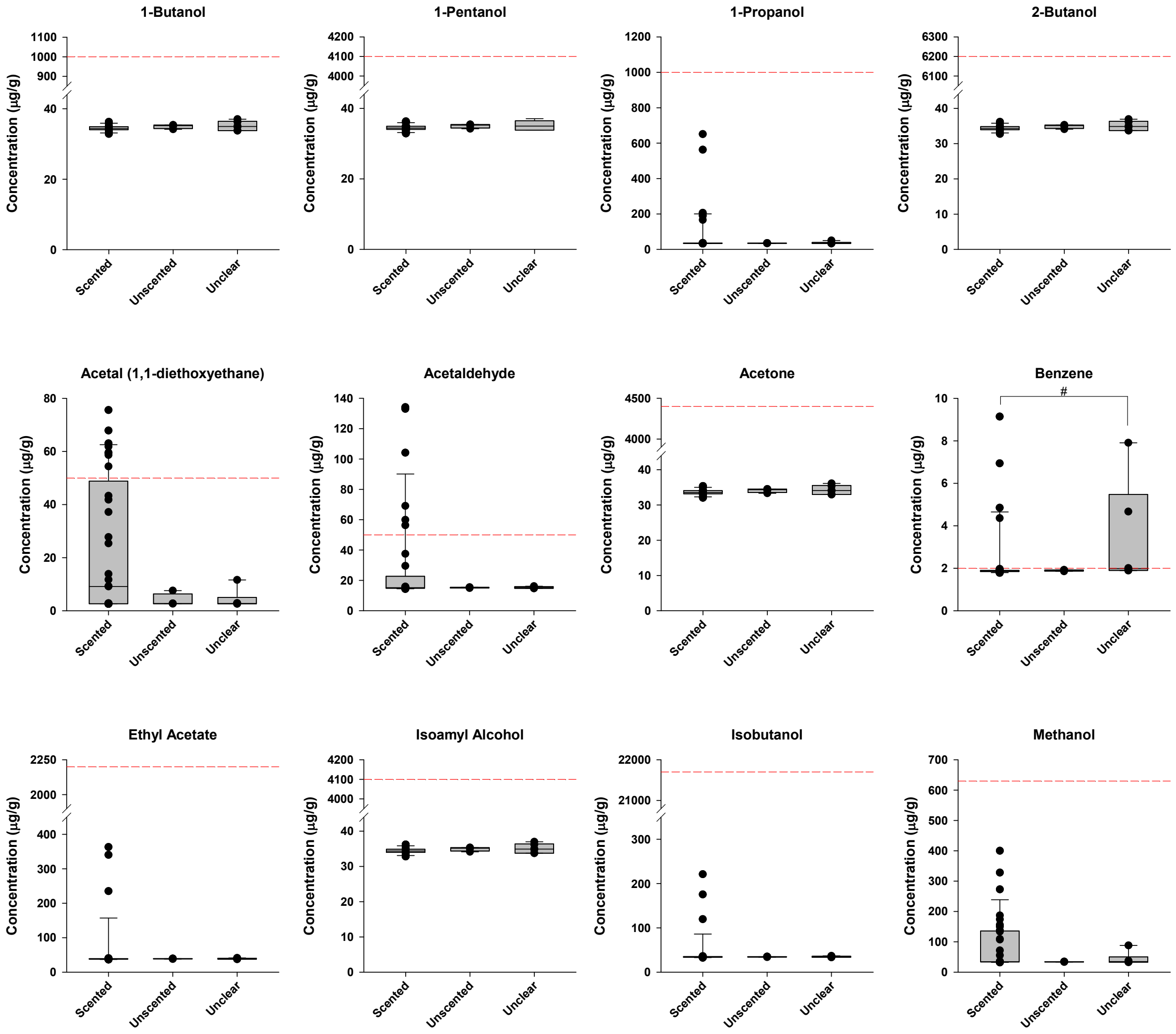

Figure S5. Impurity concentrations in scented or non-scented products. Statistical differences are indicated with a bracket and number sign. For details on statistical tests refer to Supplemental Table 2.

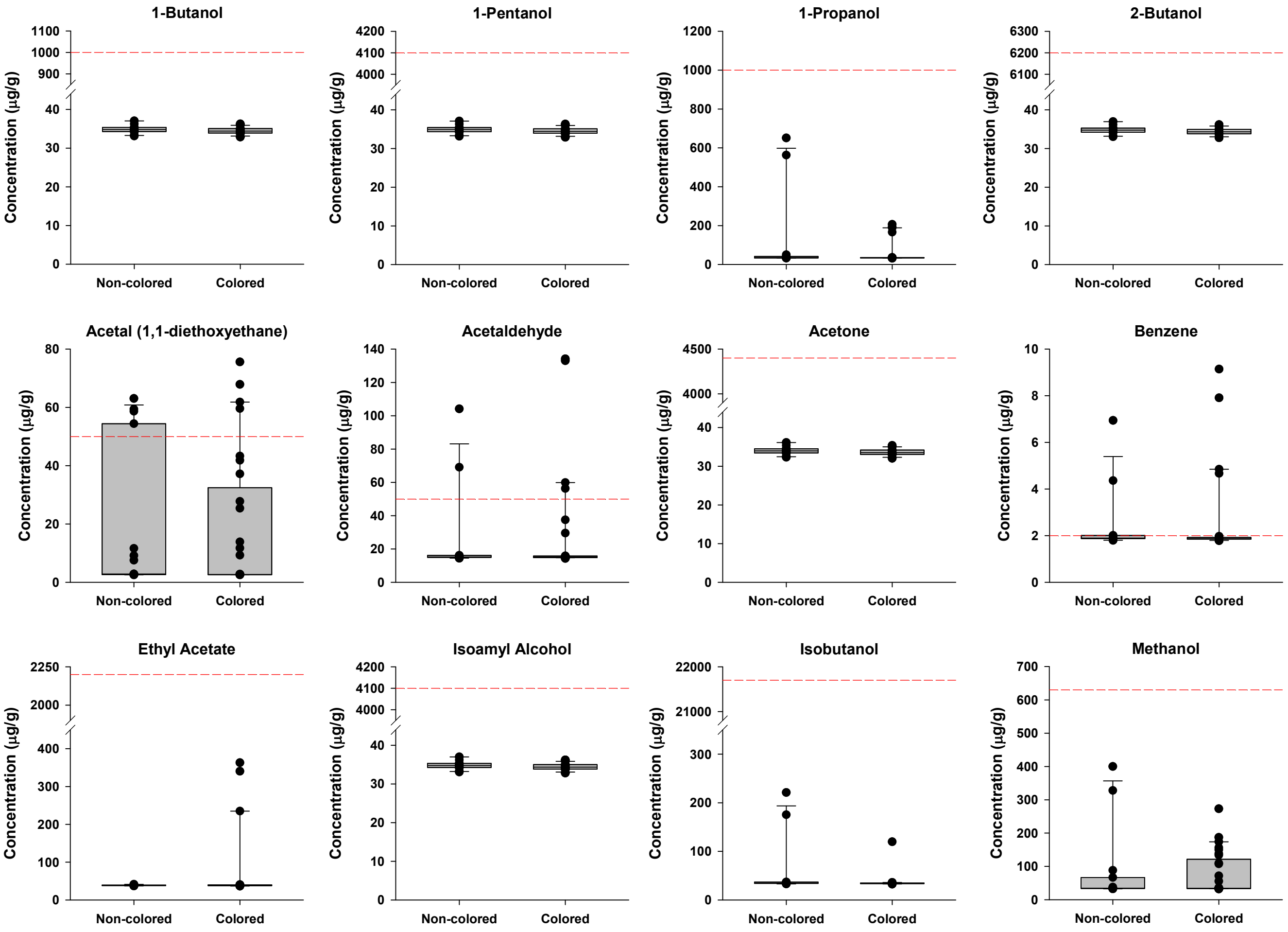

Figure S6. Impurity concentrations in non-colored and colored products. No statistically significant differences were noted. For details on statistical tests refer to Supplemental Table 2.

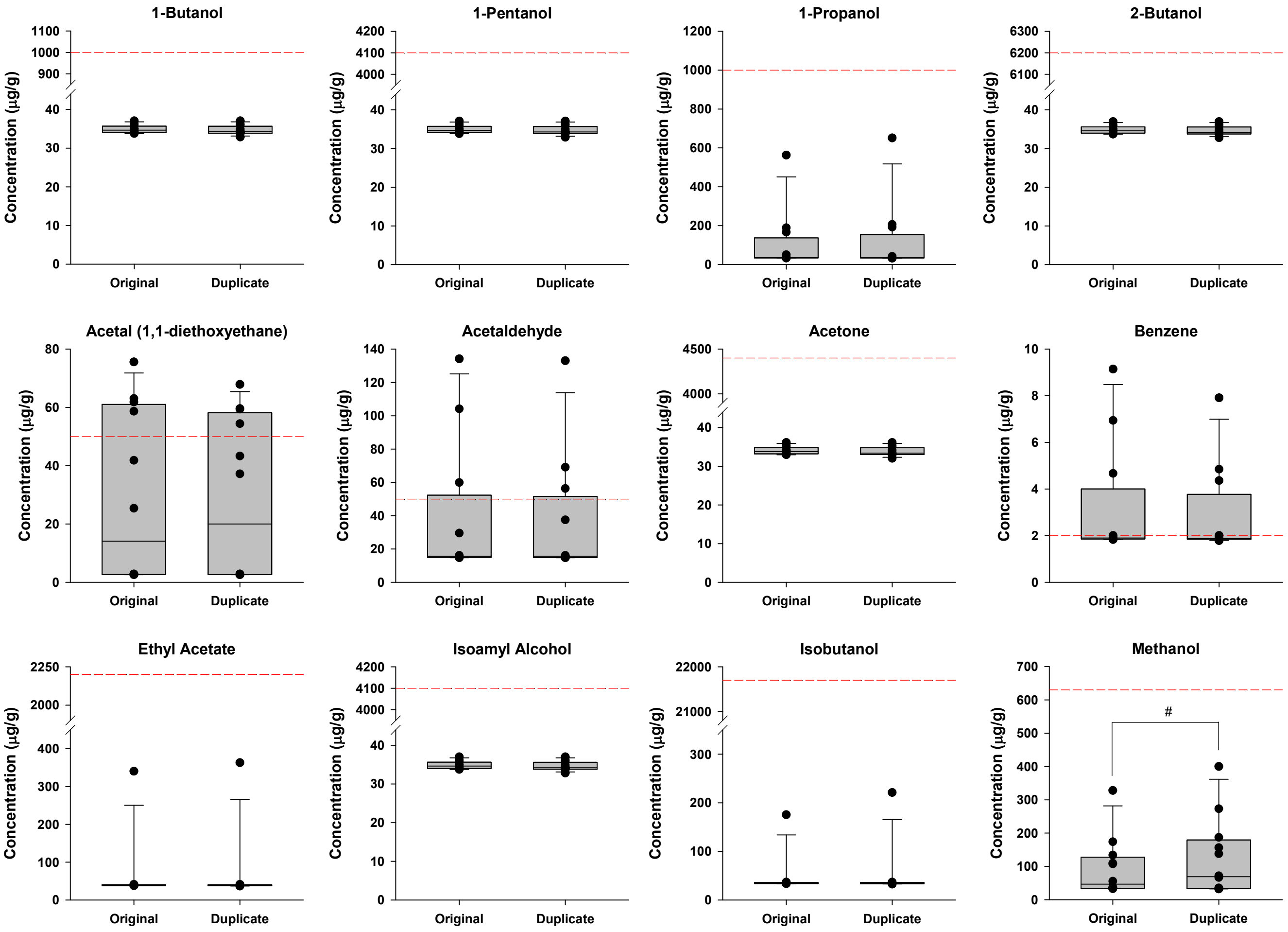

Figure S7. Impurity concentrations in original and duplicate samples. Statistical differences are indicated with a bracket and number sign. For details on statistical tests refer to Supplemental Table 2.

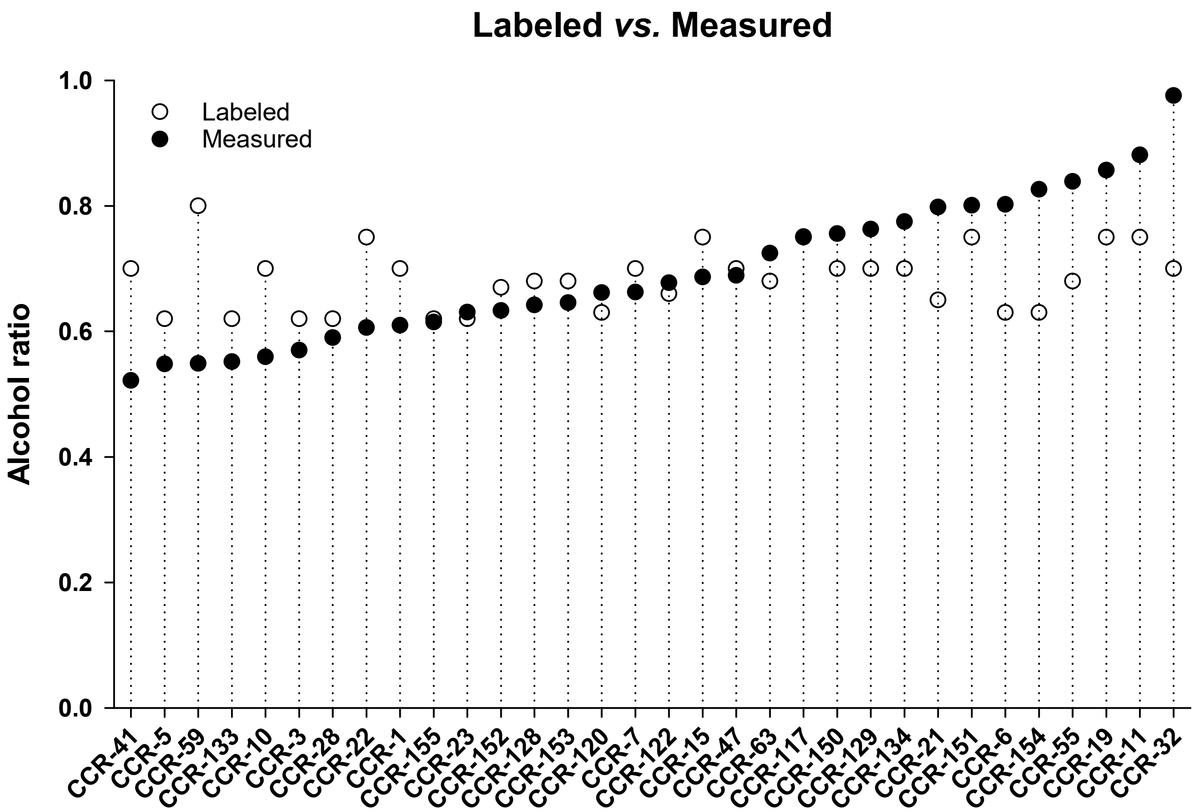

Figure S8. Labeled vs. measured alcohol concentrations in hand sanitizers marketed to children (expressed as a ratio fraction).

Table S2. Statistical tests for comparing impurity concentrations in U.S. and non-U.S. products.

Impurity	Normality (Shapiro-Wilk)		Mann-Whitney Rank Sum test		Student's t-test	
	Result	p-value	Result	p-value	Result	p-value
1-Butanol	Passed	0.203	-	-	n.s.	0.314
1-Pentanol	Passed	0.203	-	-	n.s.	0.314
1-Propanol	Failed	<0.050	n.s.	0.945	-	-
2-Butanol	Passed	0.203	-	-	n.s.	0.314
Acetal	Failed	<0.050	n.s.	0.771	-	-
Acetaldehyde	Failed	<0.050	n.s.	0.824	-	-
Acetone	Passed	0.203	-	-	n.s.	0.314
Benzene	Failed	<0.050	n.s.	0.288	-	-
Ethyl acetate	Failed	<0.050	n.s.	1.000	-	-
Isoamyl alcohol	Passed	0.203	-	-	n.s.	0.314
Isobutanol	Failed	<0.050	n.s.	0.320	-	-
Methanol	Failed	<0.050	*	***0.034***	-	-

* Concentration in non-U.S. products is significantly higher than in U.S. products; 'n.s.' denotes 'not significant'; '-' not tested.
